# Chat-based digital clinic encourages individuals at high risk of coronary heart disease to contact physician

**DOI:** 10.1016/j.ajpc.2026.101506

**Published:** 2026-02-25

**Authors:** Eero Aaltonen, Nina Mars, Sanni Ruotsalainen, Roope Ripatti, Johanna Aro, Marianne Leinikka, Iiro Heikkilä, Kristina Hotakainen, Elisabeth Widén, Samuli Ripatti

**Affiliations:** a Institute for Molecular Medicine Finland (FIMM), HiLIFE, University of Helsinki, Finland; b Broad Institute of MIT and Harvard, Cambridge, MA, USA; c Department of Public Health, University of Helsinki, Finland; d Department of Clinical Chemistry and Haematology, University of Helsinki, Finland; e Mehiläinen Oy, Helsinki, Finland

Comprehensive assessment of risk is beneficial in primary prevention of atherosclerotic cardiovascular diseases (ASCVDs), and the current clinical guidelines recommend 10-year-risk estimation in middle-aged individuals [1]. During the last decade polygenic risk scores (PRS) have been shown to improve prediction over conventional risk factors [2,3]. PRSs for coronary heart disease (CHD) can recognize at-risk individuals who may have low risk based on their conventional risk factors alone [4], and there is a growing interest in translating them to clinical practice [5].

While interventions providing polygenic risk information to individuals have been useful in encouraging some individuals for beneficial risk lowering actions, the proportion of high-risk individuals taking these actions is typically small [6,7]. For example, in our GeneRISK study we communicated 10-year-ASCVD-risk, including polygenic risk for CHD and individuals at high risk were encouraged to discuss the results with a physician. However, only 20 % of high-risk individuals had contacted a physician during the 18-month follow-up period.

In this matched cohort study, we investigated if communicating clinical and polygenic 10-year-CHD-risk through mobile devices, combined with a chat-based digital clinic, a messaging tool for contacting a physician would increase preventive actions compared to communication without such a consultation opportunity. To evaluate the effectiveness of the mobile health intervention, we used a matched control group from the GeneRISK study in which individuals received personalized risk information through a separate web-portal without chat-based access to healthcare professionals.

The digital clinic sample consisted of *N* = 804 newly recruited individuals (mean age 52.5 years, 60.4 % women) of whom 61.1 % (*N* = 491) attended the follow-up at six months. Controls (N = 2,288) were matched for age, sex, body-mass-index, systolic blood pressure, current smoking, diagnosis of diabetes, medication use and CHD-PRS. Detailed explanation on study procedures, including cohort characteristics is provided in supplementary material. Based on the individual 10-year-CHD-risk estimates, 50.7 % were at low risk (<2.0 %), 41.8 % at average (2.0–7.5 %), and 7.5 % at high risk (>7.5 %). Without having the CHD-PRS as an additional risk factor in the model, these proportions would have been 56.8 %, 39.2 % and 4.0 %, for low, average and high-risk groups, respectively. Majority of participants considered the risk information easy to understand (93.5 %; Yes vs. Neutral or No) and useful (90.8 %; Agree or Partly Agree vs. rest) when communicated through a mobile device.

After the six-month follow-up period, 12.8 % (*N* = 58/452) had lost at least 5 % of their baseline body weight, and 20.2 % (*N* = 17/84) of smokers had quit smoking. The overall physician contact rate was 20.0 % (*N* = 98/491), and of these contacts 76.5 % were chat-based consultations and 23.5 % traditional in-person appointments. Compared to the matched controls, individuals in the digital clinic group were more likely to contact a physician following digital risk communication (relative risk RR=1.8; 95 %CI 1.4–2.3; *P* = 4.8 × 10^–7^; adjusted for age, sex and 10-year-CHD-risk). The difference in contact rate was largest in the high-CHD-risk group (36.4 % vs. 18.0 %; RR=2.0; 95 %CI 1.2–3.4; *P* = 0.008), but having the digital clinic available increased contacts significantly also in the average (21.5 % vs. 11.3 %; RR 1.7; 95 %CI 1.2–2.5; *P* = 0.001) and the low risk groups (16.7 % vs. 8.4 %; RR=2.0; 95 %CI 1.2–3.2; *P* = 0.006; Fig. 1).

**Fig. 1:**
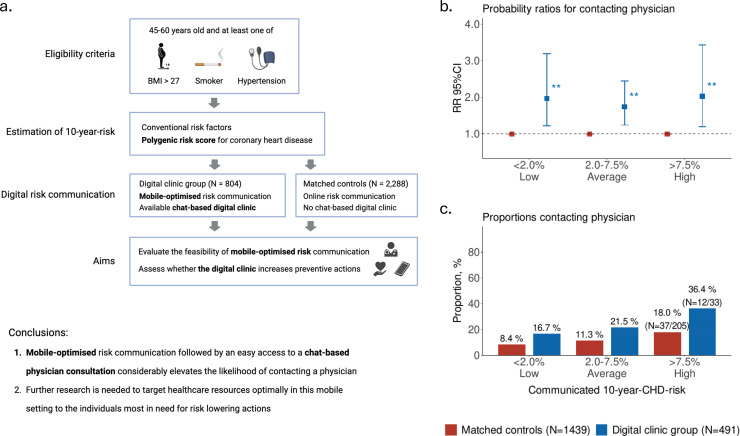
Study protocol and objectives for comparing two digital atherosclerotic cardiovascular disease prevention programs among middle-aged Finnish individuals. a. Study protocol and objectives. b. Probability ratios for contacting a physician during follow-up, with higher likelihood for detected in the digital clinic group. Adjusted RRs were derived from binary regression models fitted separately in each risk group. All models were adjusted for age, sex and 10-year-CHD-risk. c. Proportions contacting a physician during follow-up in each risk group. **P < 0.01.

In the follow-up questionnaire, participants were asked to report the outcome of the chat-based consultation if they had one. Based on these answers (*N* = 25), 48.0 % were given guidance on how to manage their lifestyle risk factors, 40.0 % were referred to an online body weight management and nutritional coaching program and 12.0 % were prescribed medications (antihypertensives, lipid-lowering medications or both) through the digital clinic.

Our result show that mobile-optimised CHD-risk communication followed by an easy access to a chat-based physician consultation considerably elevates the likelihood of contacting a physician compared to only communicating the individual’s risk. A great majority of participants found the communicated risk information easy to understand and useful.

Previously only few studies have evaluated the effectiveness of communicating composite cardiovascular disease risk estimates through mobile applications [[7], [8], [9]]. In a randomized controlled trial with *N* = 934 individuals [9], an application with multiple motivational features, including personalized risk estimates, did not increase adherence to preventive medications compared to usual care. Around 40 % of participants had existing cardiovascular disease whereas our study focused on the primary prevention in healthy population. A smaller pilot study showed promising results when incorporating risk scoring in a mobile application aimed at preventing stroke [8], and larger trials on the effectiveness of the Stroke Riskometer App are ongoing [10]. Muse et al. [7] communicated polygenic risk information alongside clinical risk scores (myGeneRank App), and individuals at high relative to low genomic risk were more likely to initiate lipid-lowering medications. Our results implicate that a simple messaging tool that is integrated with mobile risk communication could increase preventive medication and lifestyle interventions by facilitating discussions between physicians and individuals at high-risk of CHD.

There are some limitations in our study. First, the study contains middle-aged Finnish individuals, being of European ancestry and therefore limiting the generalizability of our findings to diverse ancestries or other age groups. Second, we used a quasi-experimental design to evaluate intervention effects with potential for confounding. To minimize the risk of potential confounding, we matched the control group for baseline risk factors using propensity scores and used multivariable binary regression models to calculate covariate-adjusted effect estimates. The possibility of secular trends cannot be ruled out in our study. Finally, the groups were followed for different times: the digital clinic cohort was followed for six-months compared to 18-months in the control group study and therefore we may underestimate the effect of the chat-based digital clinic.

In summary, our study shows the benefit of using an integrated risk tool to communicate coronary heart disease risk and allowing individuals for an immediate chat-based contact with a physician to discuss risk-lowering actions. Further research is needed to target healthcare resources optimally in this mobile setting to individuals most in need for risk lowering actions.

## CRediT authorship contribution statement

**Eero Aaltonen:** Writing – review & editing, Writing – original draft, Visualization, Methodology, Formal analysis. **Nina Mars:** Writing – review & editing, Writing – original draft, Methodology, Formal analysis. **Sanni Ruotsalainen:** Writing – review & editing, Methodology, Formal analysis. **Roope Ripatti:** Writing – review & editing, Data curation. **Johanna Aro:** Writing – review & editing, Supervision, Software, Project administration. **Marianne Leinikka:** Writing – review & editing, Software, Project administration. **Iiro Heikkilä:** Writing – review & editing, Project administration. **Kristina Hotakainen:** Writing – review & editing, Supervision, Conceptualization. **Elisabeth Widén:** Writing – review & editing, Supervision, Conceptualization. **Samuli Ripatti:** Writing – review & editing, Supervision, Conceptualization.

## Declaration of competing interest

We wish to draw the attention of the Editor to the following facts which may be considered as potential conflicts of interest: the authors Marianne Leinikka, Iiro Heikkilä and Kristina Hotakainen are employed by Mehiläinen Oy. Iiro Heikkilä is also a shareholder in Mehiläinen. The rest of the authors have no declarations of interest.

## Appendix Supplementary materials

Image, application 1

## References

[1] Arnett D.K., Blumenthal R.S., Albert M.A., Buroker A.B., Goldberger Z.D., Hahn E.J. (2019). 2019 ACC/AHA Guideline on the primary prevention of cardiovascular disease: a report of the american college of cardiology/american heart association task force on clinical practice guidelines. Circulation.

[2] Elliott J., Bodinier B., Bond T.A., Chadeau-Hyam M., Evangelou E., Moons K.G.M. (2020). Predictive accuracy of a polygenic risk score-enhanced prediction model vs a clinical risk score for coronary artery disease. JAMA - J Am Med Assoc.

[3] Riveros-Mckay F., Weale M.E., Moore R., Selzam S., Krapohl E., Sivley R.M. (2021). Integrated polygenic tool substantially enhances coronary artery disease prediction. Circ Genom Precis Med.

[4] Khera A.V., Chaffin M., Aragam K.G., Haas M.E., Roselli C., Choi S.H. (2018). Genome-wide polygenic scores for common diseases identify individuals with risk equivalent to monogenic mutations. Nat Genet.

[5] O’Sullivan J.W., Raghavan S., Marquez-Luna C., Luzum J.A., Damrauer S.M., Ashley E.A. (2022). Polygenic risk scores for cardiovascular disease: a scientific statement from the american heart association. Circulation.

[6] Widén E., Junna N., Ruotsalainen S., Surakka I., Mars N., Ripatti P. (2022). How communicating polygenic and clinical risk for atherosclerotic cardiovascular disease impacts health behavior: an observational follow-up study. Circ Genom Precis Med.

[7] Muse E.D., Chen S.F., Liu S., Fernandez B., Schrader B., Molparia B. (2022). Impact of polygenic risk communication: an observational mobile application-based coronary artery disease study. NPJ Digit Med.

[8] Krishnamurthi R., Hale L., Barker-Collo S., Theadom A., Bhattacharjee R., George A. (2019). Mobile technology for primary stroke prevention a proof-of-concept pilot randomized controlled trial. Stroke.

[9] Redfern J., Coorey G., Mulley J., Scaria A., Neubeck L., Hafiz N. (2020). A digital health intervention for cardiovascular disease management in primary care (CONNECT) randomized controlled trial. NPJ Digit Med.

[10] Feigin V.L., Owolabi M., Hankey G.J., Pandian J., Martins S.C. (2022). Digital health in primordial and primary stroke prevention: a systematic review. Stroke.

