# Supplementary material for: Chat-based digital clinic encourages individuals at high risk of coronary heart disease to contact physician
Source: Am J Prev Cardiol. 2026 Feb 25;26:101506. doi: 10.1016/j.ajpc.2026.101506 (PMC13084084; doi:10.1016/j.ajpc.2026.101506)
Supplement: Supplementary file 1 [file mmc1.docx]

Methods

Study setting and population

We conducted an observational cohort study with a six-month long follow-up period in Finland in 2019-2020 in collaboration with Mehiläinen Oy, a private healthcare provider. An information letter regarding the study was posted on our collaborator's website and on their social media platforms. Individuals self-referred to the study by filling out a questionnaire through Mehiläinen's consumer health application which they had to install to be able to participate. Eligible individuals were 40-60 years old with no history of myocardial infarction or stroke, and with at least one of the following risk factors for atherosclerosis: their body mass index exceeded 27, they had hypertension (systolic blood pressure measurement > 150 during the last year) or they were smokers. The decision to include at least one of these three risk factors as inclusion criteria was guided by three key considerations: 1) these factors are well-established as cardiovascular disease risk factors, 2) they are straightforward to measure and evaluate, and 3) they are modifiable, making them suitable for interventions. Eligibility was assessed through the questionnaire, and recruitment took place until N~1000 individuals who met the inclusion criteria had been registered. After enrollment, study individuals underwent laboratory examination where their total cholesterol (TC; mmol/l), low-density lipoprotein cholesterol (LDL-C; mmol/l), high-density lipoprotein cholesterol (HDL-C; mmol/l), triglycerides (TG; mmol/l) and systolic blood pressure (SBP; mmHg) were measured, and a DNA sample was taken.

Risk estimation and communication

Following the initial risk factor measurement, we estimated participants’ 10-year-risk of major coronary heart disease event using an in-house developed risk model. This model incorporates both conventional risk factors and a polygenic risk score for CHD. The estimated 10-year-risk, risk category and predicted future risk trajectory along with recommended actions was communicated to the individuals using an interactive, web-based risk communication tool (KardioKompassi) previously described in the GeneRISK study and now optimized for interpretation on a mobile device screen (Fig. 1). Individuals could access the risk communication tool through an embedded link in the app, and the risk estimation and communication did not require contact with a physician.

Chat-based digital clinic

Following risk communication, participants had the opportunity to discuss risk-lowering actions with a physician using a chat-based digital clinic interface (Fig. 1), a messaging tool for interactive discussions between patients and healthcare professionals. During these chat-based consultations, the physicians had access to the individual's CHD-risk report. After 6 months, a second questionnaire was released in the app assessing potential changes in health behavior and attitudes towards risk communication through a mobile device.

Figure 1. Components of the mobile health intervention


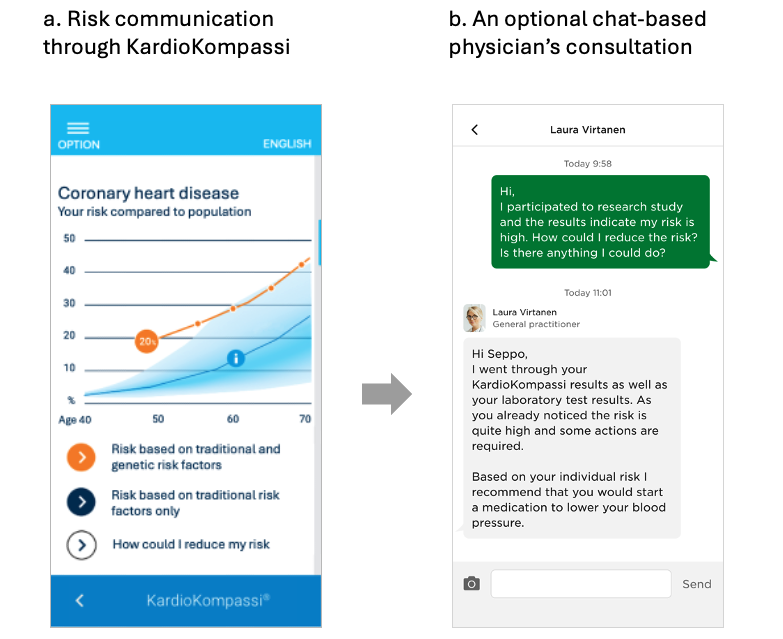


The mobile health intervention consisted of two components: First, a mobile-optimised, interactive tool (a) was used to communicate coronary heart disease risk, including polygenic risk information. In the second stage (b), participants had the opportunity to discuss risk-lowering actions with a physician using a chat-based digital clinic interface. In the control group cardiovascular disease risk was communicated through a web portal without integrated access to a chat-based physician consultation.

Matched controls

To evaluate the effectiveness of the mobile health intervention, we used a matched control group from our previous study (the GeneRISK study) in which individuals received personalized ASCVD-risk information based on conventional risk factors and a CHD-PRS through a separate web-portal without chat-based access to health professionals. The follow-up period was 18-months. Prior to matching the full digital clinic cohort was restricted to individuals aged 45 to 60 years which was the age overlap between the two cohorts. The controls were matched for age, sex, body-mass-index, systolic blood pressure, current smoking, diagnosis of diabetes, medication use and CHD-PRS using propensity scores with the nearest neighbour algorithm at a 1:3 ratio[1]. To ensure match quality, a caliper width of 0.2 SD of linear propensity score was used. Baseline characteristics of the matched cohort are provided in table 1.

Table 1. Baseline characteristics of the matched cohort

|  | Digital clinic group | Matched controls | *P*-value |
| --- | --- | --- | --- |
| N, total | N=804 | N=2,288 |  |
| Age, years | 52.5 (4.3) | 52.5 (4.2) | 0.80 |
| Sex male, % | 39.6 % | 39.9 % | 0.88 |
| Body-mass-index, kg/m^2^ | 30.7 (4.7) | 30.2 (5.4) | 0.0041 |
| Systolic blood pressure, mmHg | 137 (16) | 136 (16) | 0.46 |
| Smoking, % | 16.5 % | 16.9 % | 0.85 |
| Family history^a^, % | 27.7 % | 27.0 % | 0.72 |
| Diabetes, % | 6.0 % | 5.7 % | 0.87 |
| Antihypertensives, % | 33.7 % | 32.1 % | 0.44 |
| Lipid-lowering medication, % | 14.9 % | 14.0 % | 0.55 |
| 10-year-CHD-risk, % | 1.8 [1.0–3.3] | 1.9 [0.9–3.7] | 0.09 |
| Reported as mean (SD), proportion % or median [IQR] as appropriate.  ^a^First-degree relative with myocardial infarction before the age of 60 | | | |

Statistical analysis

The primary outcome was contact with a physician following digital risk communication which was measured through having completed a pre-appointment questionnaire in the digital clinic interface, or self-reported in-person appointment with a physician during the follow-up. From both studies, individuals participating in the follow-up were included in the primary outcome analysis. To further minimize the risk of potential confounding and derive adjusted estimates for the effect of the intervention, we used multivariable binary regression models that were adjusted for age, sex and 10-year-CHD-risk scores[2]. Secondary outcomes were changes in body weight and smoking habits during follow-up and perceived usefulness and understandability of risk information when communicated through a mobile device. The statistical analysis was conducted using R version 4.5.0, and a *P* less than 0.05 was considered statistically significant.

Ethical considerations

The study protocol was approved by the ethics committee of the Helsinki and Uusimaa hospital district (Decision No. HUS/408/2019). All study individuals gave their informed consent, and the data is pseudonymized. Study participants received no compensation for participation, but they were provided with information on their individual disease risk and had the opportunity to discuss the information with a physician through the chat-based digital clinic.

References

[1] Rubin DB. Using Propensity Scores to Help Design Observational Studies: Application to the Tobacco Litigation. Health Serv Outcomes Res Methodol 2001;2:169–88. https://doi.org/10.1023/A:1020363010465.

[2] Li X, Shen C. Doubly Robust Estimation of Causal Effect: Upping the Odds of Getting the Right Answers. Circ Cardiovasc Qual Outcomes 2020;13:E006065. https://doi.org/10.1161/CIRCOUTCOMES.119.006065.
